# Supplementary material for: Content-rich biological network constructed by mining PubMed abstracts
Source: BMC Bioinformatics. 2004 Oct 8;5:147. doi: 10.1186/1471-2105-5-147 (PMC528731; doi:10.1186/1471-2105-5-147)
Supplement: Additional File 2 — The original results of the above study (non-essential files are deleted to keep the file size under the limit set by BMC bioinformatics). [file 1471-2105-5-147-S2.bz2 › chilibotAdditionalFile2/dip05/425ID11470772E10355/html/BLR1_CCL3.html]

 


 **BLR1** and **CCL3** 
  
Found 3 abstracts in PubMed, retrieved 3.  
 

 What does Google say? 
 PDF only 
| .edu only 

---

**Interactive relationship** (e.g. stimulation, inhibition, etc)

**Neutral relationship**- However, none of a series of radiolabelled chemokines IL 8, NAP 2, GRO alpha, PF4, IP10, MCP 1, MCP 2, MCP 3, I 309, RANTES and MIP 1 alpha  [ **CCL3** ]  and other ligands C3a and leukotriene B4 bound to Jurkat transfectants that stably expressed either MDR15  [ **BLR1** ]  or  **BLR1**  mRNA.  Ref: 7639692 Biochem J, 1995
